# Adherence to daily dietary and activity goals set within a Māori and Pacific weight loss competition

**DOI:** 10.1186/s40608-019-0228-6

**Published:** 2019-03-04

**Authors:** Marewa Glover, Marrit Nolte, Annemarie Wagemakers, Hayden McRobbie, Rozanne Kruger, Bernhard H. Breier, Jane Stephen, Mafi Funaki-Tahifote, Mathu Shanthakumar

**Affiliations:** 1 School of Health Sciences, College of Health, Massey University, PO Box 89186, Torbay, Auckland, 0742 New Zealand; 2 Health and Society, Wageningen University & Research, Wageningen, The Netherlands; 3 Dragon Institute, Auckland, New Zealand; 4 School of Sport, Exercise and Nutrition, College of Health, Massey University, Auckland, New Zealand; 5 Pacific Heartbeat, Heart Foundation, Auckland, New Zealand; 6 Environmental Health Indicators Programme, Centre for Public Health Research, Massey University, Wellington, New Zealand

**Keywords:** Obesity prevention, Weight loss competition, Lifestyle challenges, Indigenous

## Abstract

**Background:**

New Zealand Pacific and Māori populations measure disproportionately high on the international body mass index (BMI). Information is needed on what behavioural weight loss goals to recommend and how to attract and retain them in interventions. Our team weight loss competition trial for participants with a BMI ≥30 used cash prizes to incentivise completion of nine daily behaviour goals. This paper evaluates the theoretical merit of and adherence to these goals.

**Methods:**

A qualitative component evaluation methodology was used. Trial data on team activity, demographics and anthropometric outcome data were extracted to determine frequency of daily goal completion by teams throughout the competition and to describe participant characteristics. T-tests were used to compare completion rates of the challenges, challenge completion by day of week and between weekdays and weekends. To examine adherence to the daily challenge activity over 24 weeks the total amount of completed challenges adjusted for number of active teams was plotted by week. A Body Shape Index (ABSI) was used to determine individual anthropometric change from baseline to 8, 16 and 24 weeks. Program documents were analysed to identify barriers to adherence and retention of participants.

**Results:**

Of 19 teams (*N* = 130) who began only five teams performed daily goals across the whole 24 weeks. Adherence was highest during the first 8 weeks. No difference in performance between goals was found suggesting they were equally viable, though tasks worth less points were performed more frequently. Goal completion was higher on weekdays. The behaviour goals appeared to have theoretical merit in that more members of high performing teams experienced a positive change in their ABSI.

**Conclusions:**

Incentives offer a promising strategy for encouraging retention in weight loss interventions. This study suggests that participants in a competition will perform incentivised tasks. The findings however, are limited by missing data and high drop out of individuals and whole teams. Further research is needed on how to increase retention.

## Background

Rates of obesity (having a BMI of 30 or over) are increasing worldwide [1]. New Zealand (NZ) has a high prevalence of obesity which varies by deprivation level and ethnicity [2]. NZ resident Pacific and Māori (indigenous) people, who are over-represented among the most deprived, have disproportionately high rates of obesity (68.7 and 50.2% respectively) compared to the European population (30.5%) [3]. These ethnic inequalities are reflected in excess incidence and mortality rates for Pacific and Māori populations in several obesity-related cancers [4] and greater prevalence of diabetes (11.4% for Pacific males and 11.6% for females; 8.2% among Māori men and 7% for females; versus 3% for NZ European/Other males and 2.2% for females) [5]. Cardiovascular disease (CVD) is experienced disproportionately also. In 2012–2014, Māori people were more than 1.5 times as likely as non-Māori people to be hospitalised for CVD [6].

Changing and controlling dietary behaviours and increasing physical activity [7–9], is the primary lifestyle treatment recommended for obesity [10]. Behavioural therapy, including techniques such as self-monitoring, stimulus control, goal-setting, problem-solving, relapse prevention, cognitive restructuring and motivation enhancement are believed to improve adherence to behavioural change interventions [11].

Although mortality associated with obesity has declined over the last three decades in NZ, the rate of decline has been slower among Māori and Pacific Island populations [12, 13]. Evidence is needed on how best to engage and retain Māori and Pacific people in weight change programmes, and what behavioural changes are effective and sustainable [14, 15].

This paper assesses the daily behavioural goals set for participants of a 24 week Pacific and Māori team weight loss competition. The *Ka Mau Te Wehi* (a Māori saying that means Awesome!) competition was designed to trigger sustainable changes that could result in reduced CVD and type 2 diabetes (T2D) risk among adults with a BMI of 30 or over. Unlike other weight loss competitions [16, 17] WEHI participants earned points not just for anthropometric changes, but for completion of daily dietary and physical activity goals (challenges). The aim was to examine adherence to the challenges throughout the competition by asking how many and which challenges were performed. We also sought to assess the theoretical merit of the daily challenges by investigating if there was any indication that completion of the challenges contributed to the desired anthropometric changes.

### The WEHI trial

The rationale and method for the WEHI trial is fully described elsewhere [18]. Briefly, Māori and Pacific adults 16 years and over with a BMI of 30 or more were invited to participate in one of three (a Māori urban, a Māori rural and a Pacific urban) 24 week long weight loss competitions between September 2016 to February 2017. Each region was to recruit seven intervention teams of 7 people (*N* = 149) and an equal number of individuals to form a control group. Data from questionnaires and measurements was collected during September 2016 – December 2017 at baseline, 8, 16, 24 and 48 weeks from the intervention group and baseline and 24 weeks from the control group. Data collection at 48 weeks from the control participants was abandoned due to insufficient enrolment of participants. Weight and waist circumference measurements (WC) were performed by research assistants and questions on nutrition literacy and behaviour, physical activity, food security and body image were self-completed. In each competition area a regional health provider was contracted and trained to recruit participants, collect data and co-ordinate the competition. They distributed recruitment notices and gave presentations through their existing networks. People who were pregnant, breastfeeding or using nicotine (due to its association with weight control [19]) were excluded.

**Fig. 1:**
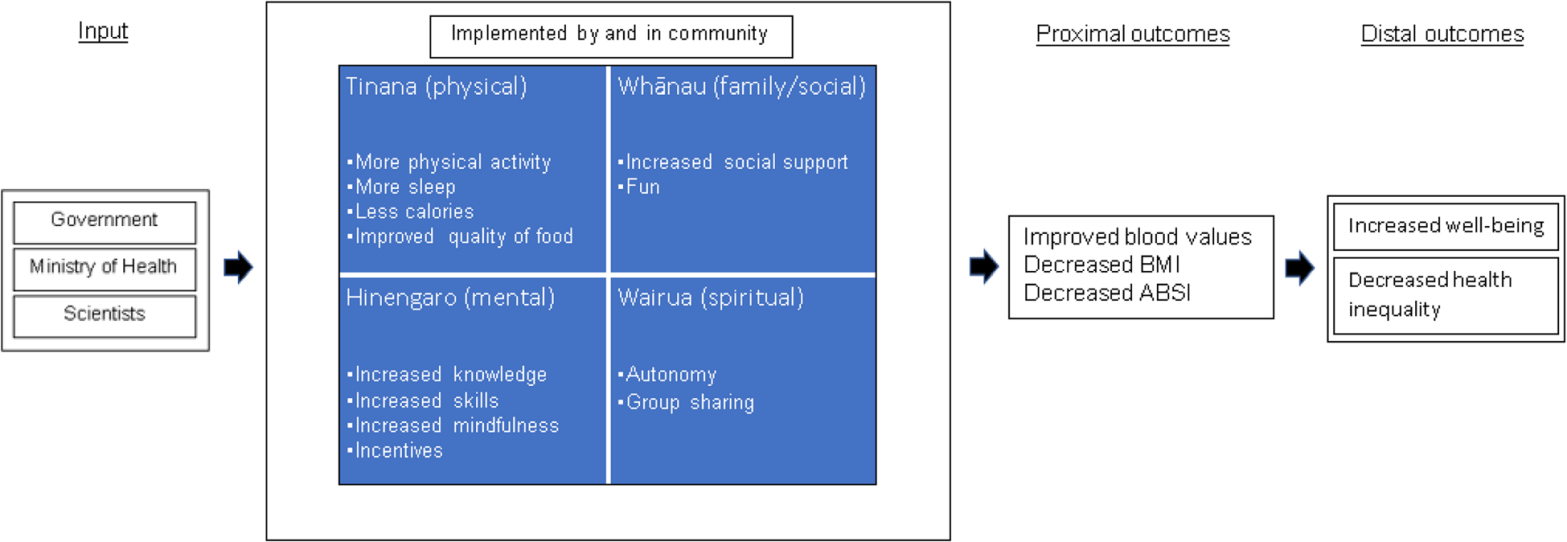
Program theory WEHI

Te Whare Tapa Wha (the four-sided house), a Māori holistic model of health underpinned the WEHI program theory (Fig. 1). Program theory explains how the elements of an intervention are expected to work and how it is expected to achieve the desired results [20]. Te Whare Tapa Wha posits that wellbeing is determined by balance across several realms: te taha tinana (physical/bodily health), te taha hinengaro (the mental realm of knowledge and emotions), te taha whānau (one’s environment including the quality of family and social relationships and social belonging) and te taha wairua (the spiritual realm). These proximal determinants of health are impacted on by the wider socio-historical-political environmental context (te ao tūroa - the long-standing environment). Pacific cultures have similar holistic health models, such as the Fonofale model, which emphasises the centrality of family and culture [21].

**Fig. 2:**
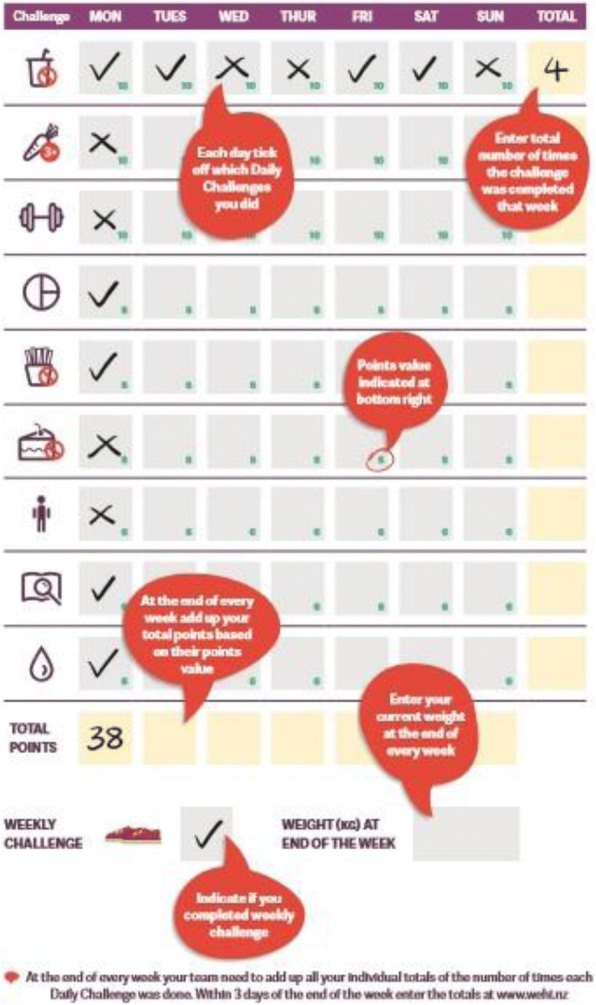
WEHI diary to record daily challenges

**Fig. 3:**
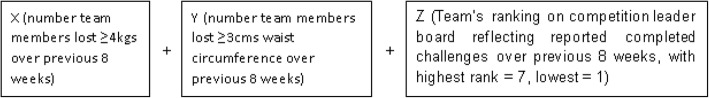
Formula used to calculate team progress at 8, 16 & 24 weeks

The four intervention components based on this theory were: 1) support from both the health provider and team members (te taha whanau), 2) within region inter-team competition with incentives (spot and cash prizes) for completing (te taha hinengaro), 3) daily and weekly behavioural challenges (te taha tinana), and 4) internet delivered education and support (te taha hinengaro). Programme resources were provided to enable individual and team activity tracking (individual activity diary (Fig. 2) and a team activity summary sheet) and reporting (via the website). Each team was given a set of bathroom scales to use as a motivational tool at group meetings if they wanted to. Each participant received a fridge magnet listing the daily challenges as a mnemonic device. At the end of week 8 and 16 the leading team in each competition, based upon team members’ weight and WC loss and highest team activity score (Fig. 3), won $NZ1000 ($US678, €587). The final prize at 24 weeks was $NZ3000 per region. The prize went to the team’s nominated charity.

### Daily challenges

The WEHI daily challenges emerged from a literature review to identify behavioural changes associated with weight loss. The *NZ Eating and Activity Guidelines for Adults* [22] were given priority to ensure consistency with Ministry of Health goals. An initial list of goals were then ranked by seven of the WEHI researchers, who have expertise in obesity prevention, nutrition, physical activity and behavioural change. The ranking criteria were 1) efficacy for achieving weight loss, 2) effectiveness for reducing risk of CVD and T2D but also 3) stimulating positive behaviour change instead of restricting behaviour*.* Several additional behavioural goals were suggested, such as eating with others. A second expanded list was ranked as above, then refined to ensure a final list of measurable activities that participants could sustain and that addressed a range of determinants of obesity. In addition, challenges had to be simple to understand and activities most participants could do. For example, though an important negative influence on weight control, refraining from alcohol was excluded because Pacific women are less likely to consume alcohol [23]. The researchers discussed the ranked challenges, reducing the list to nine which were assigned a points value proportionate to their importance for weight reduction. Assigning more points to the more important challenges was intended to motivate participants to give priority to those challenges. The final list of daily challenges (Table 1) mainly focused on te taha tinana, including physical activity and dietary changes, as well as te taha hinengaro activities that intended to change knowledge, habits and increase mindfulness.

**Table 1:** Daily challenges, ranking, description and rationale

Daily Challenge	Points value	Description	Rationale
Sugar-free Drink Day	10	A day without drinking sugar-sweetened soft drinks or fruit juices or cordials. This included not adding sugar to tea or coffee.	One of the major sources of added sugars are non-alcoholic beverages, such as sugar-added carbonated drinks and fruit juices [22]. According to the 2008/09 New Zealand Adult Nutrition Survey, 35.5% of Māori adults and 39% of NZ Pacific adults have sugary drinks three or more times each week [56]. The Ministry of Health Nutrition Guidelines recommend limiting consumption of sugar-added drinks to less than once a week [22]
3+ Vege Day	10	A day when at least 3 servings of vegetables were consumed.	Replacing high density foods with vegetables can help prevent excess weight gain since they are low in energy [57] In addition, this decreases the risk of non-communicable diseases such as stroke and some types of cancer [58, 59]. For this reason, it is recommended that New Zealanders consume at least three servings of vegetables per day [22].
Exercise Day	10	A day when at least half an hour continuous walking or more strenuous exercise was completed.	A low amount of physical exercise has been proposed as a main contributor to obesity [60]. It has been suggested that increasing physical activity may reduce obesity [61, 62].
¼¼½ Dinner Day	8	A day when the main meal is made up of ½ vegetables, ¼ protein and ¼ carbohydrates.	Healthy eating patterns involve eating a range of foods from four food groups: fruits and vegetables, grain foods, diary and legumes, chicken, nuts or red meat [22]. It is recommended that half of the meal consists of non-starchy vegetables because these are lower in total energy and contain a lot of vitamins and minerals. One quarter of the meal should consist of carbohydrates such as potato or kumara (sweet potato) and the last quarter should be protein-rich products [63].
Fast & Fried-free Day	8	A day without fast or fried food.	Fast food products are usually high in calories [64]. Reducing the intake of saturated fat or replacing it by unsaturated fats is linked to a reduced risk of CVD [65]. Reduction in the intake of fast and fried foods, which are often deep-fried and high in saturated fats, is recommended [66, 67].
Sweet Treat-free Day	8	A day without eating sugar-sweetened biscuits, cakes, lollies, chocolate or puddings.	The intake of added sugars is linked to excess weight gain. The WHO recommends lowering the intake to less than 10% of the total energy intake [22, 68].
Stand Up Day	6	A day when long periods of sitting down are broken up by standing for at least a minute and this is done at least 3 times (once in the morning, once in the afternoon and once in the evening).	Over the last few decades, the time spent sitting has increased among NZ adults [69]. There are several reasons for this, including increased use of technology, and the built environment and transport being increasingly structured around sitting [70]. Taking regular breaks to stand and move around has been recommended to counter this [71].
Water First Day	6	A day of drinking a glass of water no more than 5 min before eating each of your 3 main meals (breakfast, lunch & dinner).	This behaviour was intended to prompt mindfulness. Kabat-Zinn defines mindfulness as: ‘awareness that emerges through paying attention on purpose, in the present moment, and non-judgmentally to the unfolding of experience moment by moment’ [72]. It is proposed that mindfulness can be employed to increase awareness of what you are eating and slow down your eating. Mindfulness is associated with lower calorie intake and healthier food choices [73].
Build Me Up Day	6	A day when at least 10 continuous minutes was spent focused on learning about or practising a new behaviour that will reduce stress, improve sleep quality or improve knowledge of nutrition. Visiting the WEHI website, reading the Tip of the Day and posting on the team page can count towards this.	This challenge was included to encourage participants to visit the website where they could find extra information on losing weight via the Tip of the Day, which was a main source of educational information, motivational messages and suggestions for dealing with barriers to change.

The cumulative points earned per team per week were entered by the team into their WEHI web page. Requiring only cumulative team points was intended to reduce participant burden.

To protect the competitive element of the intervention and the value of the prize incentive, challenges were offered as a range of activities to choose from, rather than a pragmatic or sustainable plan that could be enacted fully every day. If the plan was too easy then too many teams could ‘win’, reducing the incentive value if the prize had to be divided among too many winning teams.

## Methods

A qualitative component evaluation methodology was used to assess the viability of the WEHI daily challenges. Component evaluation focuses on a particular aspect of an intervention to test and validate a program’s theory [24]. Of interest is whether or not the component was implemented as intended and adhered to and if so, whether the proximal outcomes aimed for could be detected and thus could indicate that the program theory was feasible [24, 25]. This then informs the process to test the generalisability of the program, that is, if the aspect under investigation is likely to work once transferred to other sites or providers [25].

Demographic and outcome data were drawn from the WEHI trial data to calculate frequencies and describe participant characteristics. Program documents were analysed to identify barriers to adherence and retention of participants. Document analysis is commonly used in program evaluations to corroborate or help explain quantitative results [26].

### Data analysis

Team activity data was downloaded from the website and demographic and anthropometric data was extracted from the WEHI trial database and entered into Excel [27]. The daily challenge data were used to calculate descriptive statistics and frequency counts of daily challenge activity. T-tests were used to compare completion rates of the challenges, challenge completion by day of week and between weekdays and weekends. To examine adherence to the daily challenge activity over 24 weeks the total amount of completed challenges adjusted for number of active teams was plotted by week. Adjustment was done by dividing the total amount of completed challenges by the number of active teams each week. To examine the theoretical merit of the daily challenges we hypothesised that more members from teams with higher total points at 8, 16 and 24 weeks would experience positive anthropometric changes. We first compared the active teams on gender, age and baseline BMI to test for differences.

A Body Shape Index (ABSI), introduced in 2012, is a new body index that detects an association between body composition and all-cause mortality more reliably than BMI and WC [28]. ABSI (WC/(BMI2/3 x height½)) [29] was used to determine individual anthropometric change from baseline to 8, 16 and 24 weeks because participants were encouraged to exercise daily which could potentially increase muscle mass reducing detection of loss in body fat in the short-term even though risks may be reduced [29, 30]. Anthropometric change over each of the three 8-week competition periods, was calculated by subtracting ABSI of the participant at the beginning of the 8-week period from ABSI at the end of that period. A positive difference suggested a reduction in WC or weight, whilst a negative difference indicated an increase. Individual ABSI difference was then plotted against the team’s cumulative daily challenge score for each 8 week period. Scatterplots are often used as a first step to explore if a likely correlation exists between variables [31]. The most this exploratory method can do, however, is provide an indication of program theory feasibility. Tests for an association between adherence to daily challenges and anthropometric change were not possible because only cumulative team performance, not individual performance, of daily challenges was collected.

Using a deductive analysis approach [26] WEHI documents were searched for information on how the programme was implemented. Monthly reports from regional co-ordinators (*N* = 10), monthly reports to the funder (*N* = 4) and emails between the regional co-ordinators and the research team were identified as most useful. These were read to identify and categorise reported barriers to adherence and implementation.

## Results

In total, 130 participants (17 teams of 7, 1 team of 6 and 1 team of 5) began the intervention. Participants were mainly female (82%), 63% were Māori, 42% were aged 45–54 years (*n* = 55). Half of the participants had some education beyond secondary school and most (79%) were currently employed (Table 2). Mean BMI at baseline was 41.0 (kg/m^2^) (range 30.3–59.4).

**Table 2:** Demographics (*n* = 130)

	Count	%	
Ethnicity			
Māori	81	63%	
Pacific	46	35%	
Pākehā	3	2%	
Age			
Under 35	24	19%	
35–44	26	20%	
45–54	55	42%	
55–64	21	16%	
65–74	4	3%	
Sex			
Male	23	18%	
Female	107	82%	
Highest completed qualification			
School	52	40%	
Post-school	72	55%	
Missing	6	5%	
Work situation			
Working	104	80%	
Not working	20	15%	
Missing	6	5%	
	Mean	SD	Range
Weight (kg)	113.3	21.2	73.5–176.4
Height (m)	1.66	0.07	1.49–1.89
Waist circumference (cm)	118.3	14.2	92.2–164
BMI	40.9	6.30	30.3–59.4

**Fig. 4:**
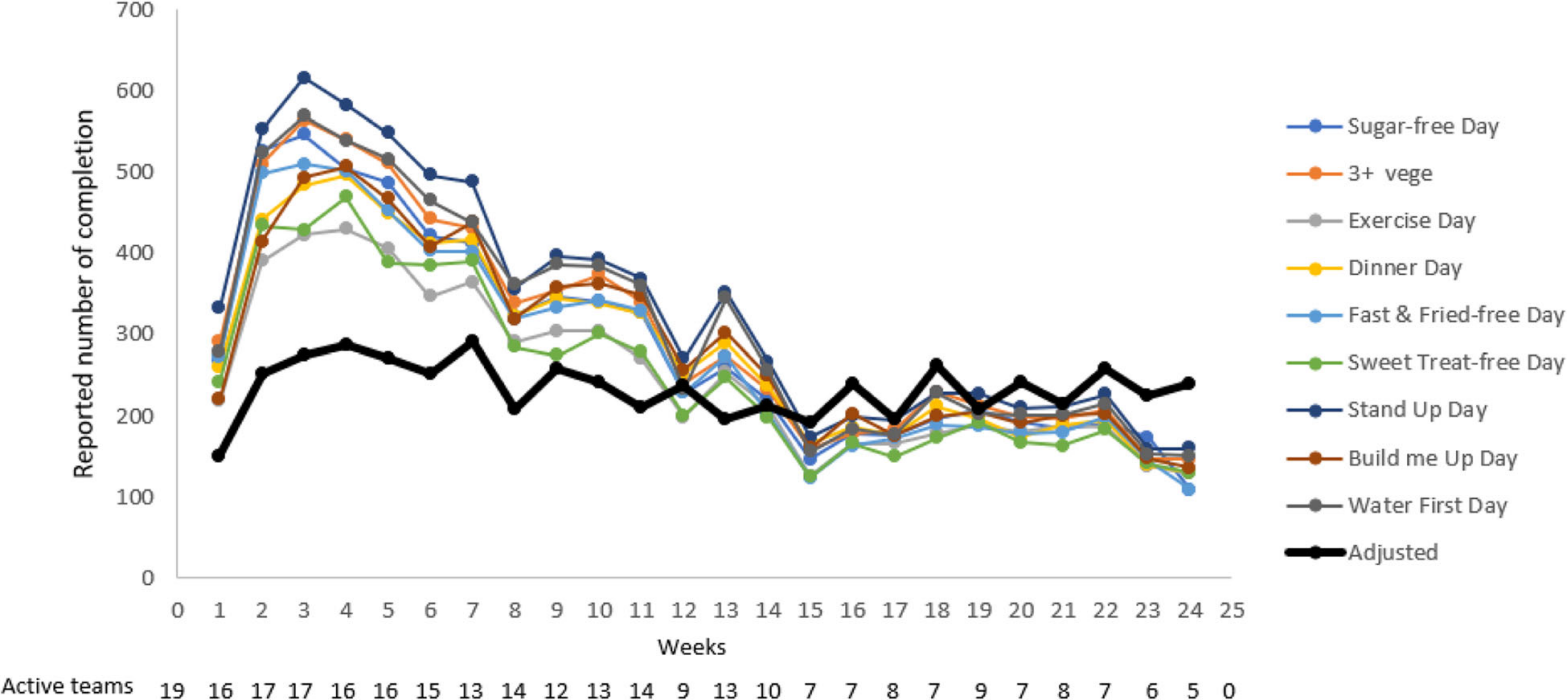
Number of completed daily challenges over the 24 weeks (coloured lines) and average number of completed daily challenges adjusted for active teams (black line)

Adherence was highest during the first 8 weeks of the programme (Fig. 4). After that the reduction in total reported challenges reflects the drop out of participants and or whole teams. The number of active teams decreased from 19 active teams in the first week to five active teams at the end of the competition. Most of the Northland teams dropped out after 15 weeks. Five participants withdrew or dropped out due to pregnancy or personal commitments. After adjusting the reported challenges for active teams, the drop off in completed challenges was less significant. Active teams reported a steady completion of daily challenges.

**Fig. 5:**
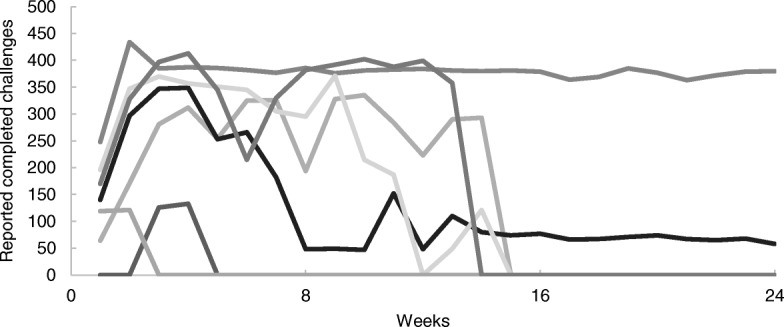
Number of completed challenges per team per week (Northland) (maximum completion = 441)

**Fig. 6:**
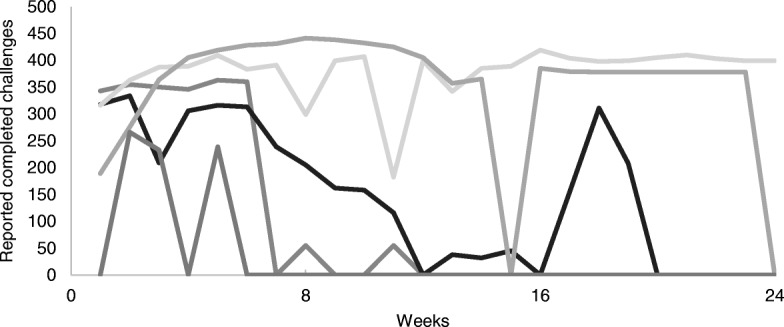
Number of completed challenges per team per week (Auckland (Pacific)) (maximum completion = 441)

Proportion of daily challenges completed each week by team against a maximum of 441 if all team members performed every challenge every day of the week is shown in Fig. 5, 6, 7 (1 per region). Daily challenge activity fluctuated widely within teams. The highest standard deviation (SD) within a team per week was 185. Some teams did not complete any challenges for 1 week but did enter completed challenges the week after. Throughout the competition, the active teams earned on average 234 (min = 150, max = 288) points for completing challenges per week. The winning teams in each region completed an average of 376 (Northland), 378 (Auckland) and 213 (Manawatu) challenges per week. In Northland and Auckland, the winning teams were quite steady in reporting challenges with no significant difference between them.

**Fig. 7:**
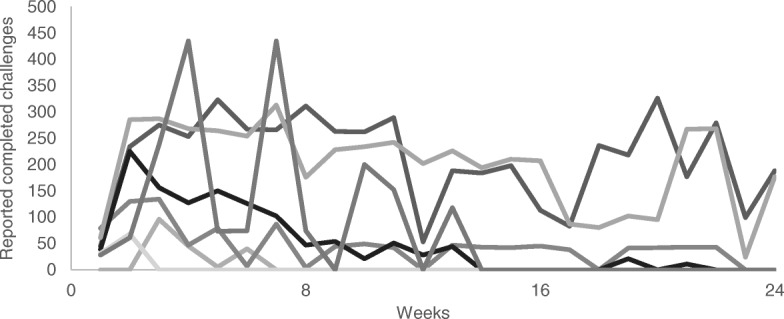
Number of completed challenges per team per week (Manawatu) (maximum completion = 441)

**Fig. 8:**
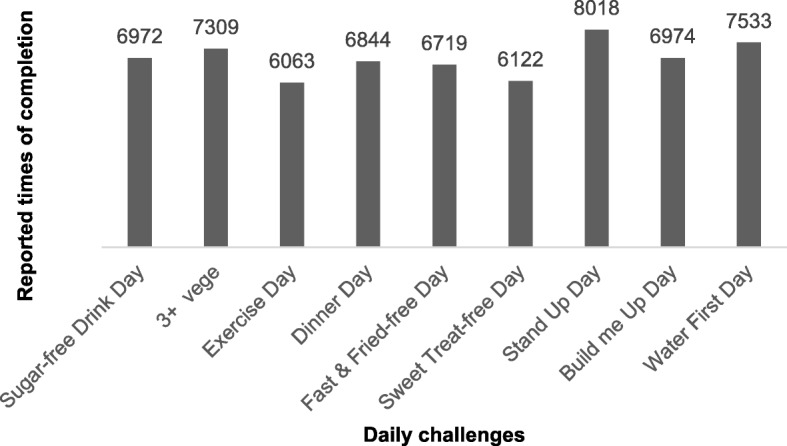
Frequency of daily challenges (maximum completion = 22,344)

Stand Up Day was completed most frequently followed by Water First Day (Fig. 8). Exercise Day and Sweet Treat-free Day were performed the least. Regional co-ordinator reports noted that participants found the Exercise Day and Sweet Treat-free Day the hardest. The easiest challenges to perform were reportedly Stand Up Day and Sugar-free Drink Day. The lowest ranked challenges (worth 6 points each) were in total performed more frequently than the highest ranked challenges (worth 10 points each) though this difference was not significant (*p* = 0.2).

**Fig. 9:**
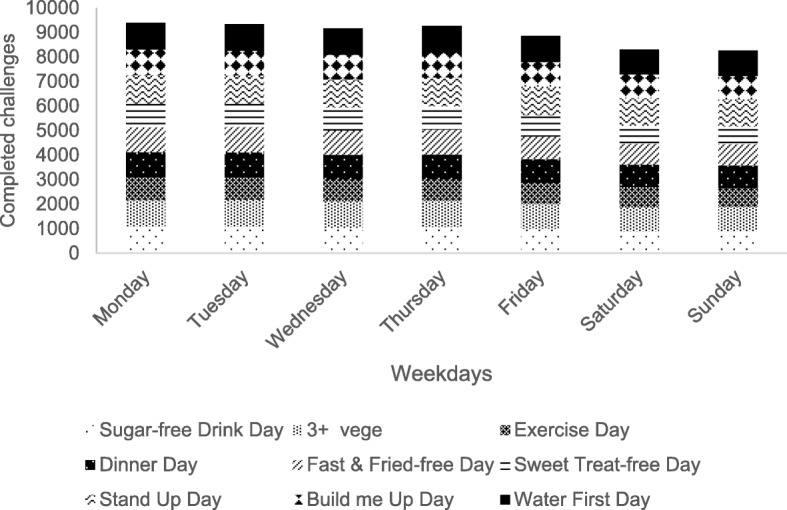
Reported daily challenges per day of the week

Challenges were more likely to be completed on weekdays than on weekends (Fig. 9), with significant differences for the Sugar-free Drink Day (*p* = 0.00), 3+ Vege Day (*p* = 0.006), ¼¼½ Dinner Day (*p* = 0.002), Fast & Fried-free Day (*p* = 0.004), Sweet Treat-free Day (*p* = 0.000) and Stand Up Day (*p* = 0.018).

Reported barriers to adherence included physical injury, drop off in motivation, lack of social support and competing priorities. For example, one of the participants injured her ankle and was not able to exercise.

**Fig. 10:**
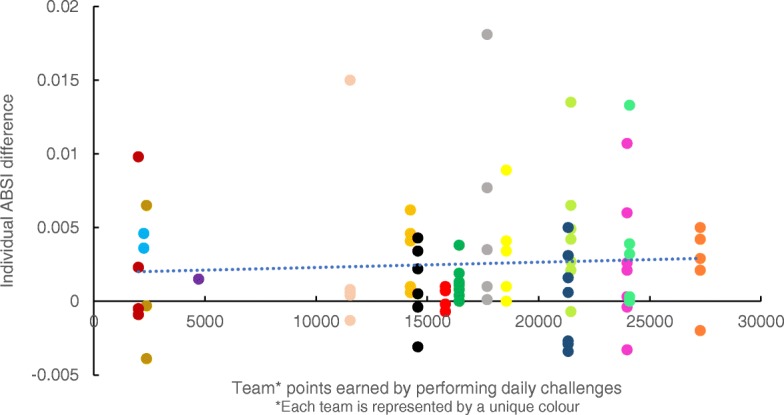
Individual difference in ABSI plotted against team points achieved in week 0–8 (*n* = 85). Included participants had full data on weight, waist, height at baseline and 8 weeks and were part of a team which earned a significant amount of team points during this period

Figure 10 suggests that the higher the teams’ total points, the more likely their members experienced a positive change in ABSI. This pattern was not obvious for weeks 8–16 and reversed for week 16–24 (data not shown) as the number of participants and active teams dropped off.

## Discussion

This study evaluated the key behavioural component of a culturally-informed team weight loss competition by assessing reported team adherence to the daily challenges and the feasibility that performance of these might result in positive anthropometric changes. Prevalence of obesity-related disease is rising and the efficacy of interventions to reduce CVD/T2D risk factors such as obesity remains contested [32]. Meanwhile, health systems are under pressure to achieve efficiency gains [33]. Evaluations of programmes to assess if they were implemented as intended are important, however, they often omit validation of the behavioural change components against the program theory [24]. Assessing the integrity with which a program is implemented becomes less useful if the behavioural change component of a programme is ineffective.

Retention of participants in this trial was poor. However, high drop-out rates and decreasing adherence in weight loss interventions is common [34–36]. Adherence to the WEHI daily challenges decreased over time and drop out of teams past the first 8 weeks limited analysis. Only the five teams who won money at 8 or 16 weeks persisted to 24 weeks and only four of them provided full anthropometric and daily challenge completion data. Numerous barriers to retention were cited, such as, declining motivation, lack of social support, lack of time and lack of being able to prioritise involvement in the WEHI intervention. Most of the participants were employed. The required activity tracking, necessity to compile team activity data each week and enter it online compounded what was already an intensive intervention requiring participants to plan for and complete up to nine challenges each day. Participant burden may have been too high for all but the winning teams leading to reduced adherence in reporting and subsequent loss of motivation to perform the challenges [37]. Adherence was significantly lower on weekends. Other studies have reported a similar pattern, for instance, American adults reportedly consume more fat in the weekend [38]; meal sizes were also larger on the weekend [39] and rates of physical activity decreased [40]. Church commitments, which are an important part of Pacific culture [41], could have contributed to reduced adherence during the weekend for Pacific participants. Further research is needed to determine the attitudes and barriers to weight loss plan enactment on weekends. It has been suggested that fun leisure-time physical activities (e.g. swimming, walking) are preferred over activity integrated into daily life (e.g. standing up more or taking the stairs), or health-related fitness activity (e.g. strength-building gym classes) [34]. Contrary to this, our easy standing challenge was performed most frequently. Future weight loss competitions should differentially incentivise week days and weekend adherence, and fun leisure-time activity.

### Boosting motivation

Reduced motivation was reported as another barrier to adherence. Literature suggests that people with obesity typically engage less in physical activity and have greater motivation to avoid exercise [42]. Embedding flexibility as to which behaviours participants choose to perform has however been found to increase plan enactment [34]. Motivation appears influential in that highly specified action plans can be effective when individuals are highly motivated [34]. It was a limitation that motivation was not measured in the WEHI trial. On the other hand, WEHI participants were recruited from a general population versus a clinical population advised to seek treatment for obesity and thus were likely to be more motivated. To boost motivation, WEHI used competition and incentives.

Financial incentives have been found to be motivating [43] but as might be the case in this study, the converse could also be true that once the prize has gone to others or the chance of winning seems impossible due to high points achieved by leading teams or when treatment expectations are too high, motivation to continue wanes [43]. Retention might be improved if all participants and or their team ‘wins’ some of the prize pool, for example, proportionate to their team points. Proportioning reward to effort has been successfully used before in weight loss competitions [44]. Further studies are needed to identify the optimal structure for rewarding progress in weight loss competitions.

### The daily challenges had merit

Active teams submitted a large amount of data enabling a detailed look at the viability of the daily challenges. For the teams that completed the competition, the nine challenges appeared to be equally viable. The differential points value of the challenges was intended to motivate performance of the higher ranked behaviours. A greater differential in points should be employed to encourage greater adherence to the more important behaviour goals. It should be noted however, that the five highest performing teams, who won money at early progress points in the competition, reported very high team completion of all nine challenges most days of the week and for most of the competition. This could have undermined the detection of viability differences between the nine challenges. Teams aiming for exceptionally high challenge completion rate potentially created problems that undermined the effectiveness of the intervention. Firstly, unfeasible or non-viable behavioural change plans are hard to sustain [43] and can lead to reduced progress [35], loss of self-efficacy and delayed or curtailed motivation to try again [45]. It is highly likely that the absence of progress de-motivated participants of non-winning teams [35]. Additionally, for weight loss interventions to be effective, prescribed goals need to be acceptable to participants, able to be completed by them and congruent with contextual demands in their lives [34]. Proportional reward for effort could mitigate these reasons for withdrawal.

The lack of individual daily challenge data and the drop out of teams prevented testing for a correlation between daily challenge completion rate and intended anthropometric change. A simplistic examination of the data supported the idea that higher completion of daily challenges would plausibly lead to a positive change in ABSI, but this was only observed at the end of the first 8 weeks during which retention and adherence was highest. The daily behavioural goals were based on weight loss intervention literature and thus it makes sense that high adherence repeated over time should assist weight and WC loss. For example, the look AHEAD study, an 8 year weight loss intervention with 5145 obese adult participants, found that weight-maintenance activities such as higher levels of physical activity and reduced calorie intake were associated with increased weight loss [46]. Additionally, other literature supports that better attendance and completion is associated with weight loss outcomes. Carels et al. recruited 44 obese women to participate in a 6-month weight loss competition increasing physical activity and decreasing energy and fat intake [47]. They found a significant relationship between poor attendance and weight loss (*P* < 0.01). The greater positive anthropometric change indicated among team members in the highest performing teams, coupled with the apparent viability of the daily challenges and that high adherence and retention was achieved for teams with a high probability of winning a substantial cash prize suggests that WEHI is likely to be theoretically sound and is thus worthy of further development and testing with a larger sample size. Further evaluation assessing implementation and the barriers to adherence and retention is also warranted.

### Limitations and strengths

There were several limitations to this study. First, the drop out of participants and teams limited the amount of data available for analysis and weakened the power to test for differences and relationships. A major contributor to the drop out was that the trial ran through the NZ summer Christmas/New Year holiday period (competition weeks 16–19 for Northland and Manawatu, and weeks 12–15 for Auckland). In NZ, community health organisations typically close down over this period. This most likely contributed to the loss of momentum and subsequent lack of interest in restarting after the holiday period. Running weight loss research or interventions through an extended holiday period is not pragmatic. Apart from this timing error, lack of motivation was given as an explanation for poor adherence and high drop-out. It is a limitation of the study that motivation to lose weight was not measured. Further research is needed on the role of motivation, and how to sustain motivation, in weight loss competitions. A significant limitation was that individual challenge completion data was not collected limiting the analyses that could be done. Only cumulative team completion was data was requested to reduce participant burden. The document analysis suggested that WEHI was experienced as requiring a significant and often prohibitive amount of time. Further work is needed to identify ways to reduce participant burden while also gathering sufficient information to enable robust evaluation. Phone based apps using push notifications to request data should be explored with internet and app literate groups [48].

A further limitation was that WEHI relied on self-reported activity which is vulnerable to social desirability bias, that is, participants would have been inclined to over-report completion of goals [49]. There is a higher risk of this occurring when incentives are involved [50]. This may have been moderated in WEHI because the cash prize went to a charity chosen by the winning team rather than to team members. On the other hand, participants could also have not reported their data because the burden was too high or they forgot about it. Self-report is supported by the literature however as it is a proven way to facilitate an individual’s awareness of their behaviour which facilitates weight loss [51, 52]. WEHI did not rely on self-report of anthropometric measurements, which was a strength but different researchers and regional co-ordinators conducted the measurements in the different regions. Requiring participants to attend ‘weigh-ins’ increased participant burden and likely contributed to missing data.

It is a strength that the study focused on people with a BMI of 30 and over. Motivation to change dietary and activity levels varies by risk status and obesity class [42]. Different interventions, varying in intensity and messages are likely to be needed depending on the motivation to change. The WEHI daily challenges were sufficiently flexible to allow for this variation between team members. The flexibility to form their own teams with family and friends is another strength since social support is positively related to weight loss [53]. The use of flexibility and the team environment are also supported by Deci & Ryans self-determination theory, representing autonomy and relatedness [54]. The third component of self-determination theory, competence, is reinforced by points. Participants were predominantly females and employed limiting extrapolation of the findings to males and lower socio-economic or unemployed people who are obese. It is a strength that the study focused on a high risk indigenous group and a minority migrant population who have among the highest rates of obesity in the world.

## Conclusion

To our knowledge this is one of the first studies trialling a team-based competition with a high CVD/T2D risk indigenous and Pacific population. The high recruitment rate and retention of at least the teams who received incentives supports that team competition is an attractive health promotion strategy to prompt a focus on weight loss for Māori and Pacific participants. Further research to determine the optimal incentive structure, such as proportioning reward to effort, to boost adherence to behavioural change goals whilst also retaining more teams for a longer term is needed.

Many health systems grappling with rising obesity rates are looking for simple effective weight loss messages that can be given to individuals, delivered to groups and their communities. As both a community based and internet-delivered programme, WEHI could be scaled up to assist people to lose weight and thus reduce CVD and T2D risk delivering population health benefits. This study suggests that a team based, culturally grounded approach was attractive to a high risk group and that the key behavioural change component had theoretical merit. Future weight loss competitions should attempt to reduce intervention burden on participants, especially if targeting low SES groups who experience disproportionately poorer access to health care due to cost, lack of transport and lack of childcare [55]. Maximising support to motivate participant retention is important. Incentives offer a promising strategy for encouraging retention in weight loss interventions.

## Abbreviations

ABSI: A Body Shape Index
BMI: body mass index
CVD: cardiovascular disease
NZ: New Zealand
SD: standard deviation
T2D: type 2 diabetes
US: United States
WC: waist circumference
